# Bladder Wall Stiffness after Cystectomy in Bladder Cancer Patients: A Preliminary Study

**DOI:** 10.3390/cancers15020359

**Published:** 2023-01-05

**Authors:** Sara Monteiro-Reis, João P. S. Ferreira, Ricardo A. Pires, João Lobo, João A. Carvalho, Rui L. Reis, Renato Natal Jorge, Carmen Jerónimo

**Affiliations:** 1 Institute of Science and Innovation in Mechanical and Industrial Engineering (INEGI), 4200-465 Porto, Portugal; 2 Cancer Biology and Epigenetics Group, Research Center of IPO Porto (CI-IPOP)/RISE@CI-IPOP (Health Research Network), Portuguese Oncology Institute of Porto (IPO Porto), Porto Comprehensive Cancer Centre (Porto.CCC), 4200-072 Porto, Portugal; 3 Department of Mechanical Engineering, Faculty of Engineering, University of Porto (FEUP), 4200-465 Porto, Portugal; 4 3B’s Research Group, I3Bs—Research Institute on Biomaterials, Biodegradables and Biomimetics, University of Minho, Headquarters of the European Institute of Excellence on Tissue Engineering and Regenerative Medicine, 4805-017 Guimarães, Portugal; 5 ICVS/3B’s—PT Government Associate Laboratory, 4805-017 Braga/Guimarães, Portugal; 6 Department of Pathology, Portuguese Oncology Institute of Porto, Porto Comprehensive Cancer Centre (Porto.CCC), 4200-072 Porto, Portugal; 7 Department of Urology, Portuguese Oncology Institute of Porto, 4200-072 Porto, Portugal; 8 Department of Pathology and Molecular Immunology, School of Medicine and Biomedical Sciences, University of Porto (ICBAS-UP), 4050-313 Porto, Portugal

**Keywords:** bladder cancer, atomic force microscopy, biomechanical properties, tissue stiffness

## Abstract

**Simple Summary:**

In this work, we observed that the presence of a tumor alters the stiffness of the bladder wall in bladder cancer patients, which may indicate that this factor has an impact on the progression of the disease.

**Abstract:**

Bladder cancer (BlCa), specifically urothelial carcinomas, is a heterogeneous disease that derives from the urothelial lining. Two main classes of BlCa are acknowledged: the non-muscle invasive BlCa and the muscle-invasive BlCa; the latter constituting an aggressive disease which invades locally and metastasizes systemically. Distinguishing the specific microenvironment that cancer cells experience between mucosa and muscularis propria layers can help elucidate how these cells acquire invasive capacities. In this work, we propose to measure the micromechanical properties of both mucosa and muscularis propria layers of the bladder wall of BlCa patients, using atomic force microscopy (AFM). To do that, two cross-sections of both the macroscopically normal urinary bladder wall and the bladder wall adjacent to the tumor were collected and immediately frozen, prior to AFM samples analysis. The respective “twin” formalin-fixed paraffin-embedded tissue fragments were processed and later evaluated for histopathological examination. H&E staining suggested that tumors promoted the development of muscle-like structures in the mucosa surrounding the neoplastic region. The average Young’s modulus (cell stiffness) in tumor-adjacent specimens was significantly higher in the muscularis propria than in the mucosa. Similarly, the tumor-free specimens had significantly higher Young’s moduli in the muscularis propria than in the urothelium. Young’s moduli were higher in all layers of tumor-adjacent tissues when compared with tumor-free samples. Here we provide insights into the stiffness of the bladder wall layers, and we show that the presence of tumor in the surrounding mucosa leads to an alteration of its smooth muscle content. The quantitative assessment of stiffness range here presented provides essential data for future research on BlCa and for understanding how the biomechanical stimuli can modulate cancer cells’ capacity to invade through the different bladder layers.

## 1. Introduction

Bladder cancer (BlCa) is one of the most common malignancies worldwide, ranking 10th in incidence, and is the second most frequent urological malignancy after prostate cancer [1,2,3]. Incidence in men is roughly 3–4-fold higher when compared to women. Although BlCa can occur at any age, its diagnosis is more frequent in patients with ~70 years old, suggesting a higher risk in the elderly. In addition, multiple recurrences and consequent additional treatment are very common, making BlCa one of the costliest neoplastic diseases to treat [4]. These tumors are usually subdivided into non-muscle invasive bladder cancer (NMIBC), accounting for approximately 70–75% of newly diagnosed BlCa, and muscle-invasive bladder cancer (MIBC), with a very distinct disease progression and prognosis [5,6]. Whereas NMIBC represents a more confined disease, with a better initial prognosis, MIBC cases have a considerable aggressive behavior, with a much higher risk for spreading and metastization. Indeed, tumors that invade the muscularis can progress to higher stages in ~40% of the cases and very likely recur (~80%) [4]. Due to this fact, if not early diagnosed, MIBC may ultimately lead to morbidity and death. Moreover, even for patients who do not present metastasis at diagnosis, the standard treatment for localized MIBC is a radical cystectomy—e.g., all bladder removal—a surgery that entails severe comorbidities [7,8].

The starting point of metastasis requires cells to invade the basement membrane and migrate towards and into the vasculature, and then settle in the secondary site. Epithelial-to-mesenchymal transition (EMT), a reversible biological process in which polarized epithelial cells undergo various mechanical and molecular changes to obtain a more invasive and mesenchymal phenotype, can promote tumor growth, invasion, and intravasation [9].

Changes in the stiffness of the extracellular matrix (ECM) and thus changes in the mechanical stimuli can trigger a mesenchymal-like behavior of the cells [10]. The presence of collagen, typical in high-grade cancers (termed “desmoplasia”), is suggested to promote the progression of NMIBC to MIBC and is likely to play a role in BlCa recurrence after surgery [11,12]. Thus, distinguishing the specific microenvironment that cells experience between the mucosa and muscularis layers and how it changes with disease progression can help us understand how cells invade the muscularis propria layer, a critical hallmark for disease invasiveness. We hypothesize that the mechanical properties of each layer are distinct, and further change in the presence of tumors. The urothelium works as a capacitance layer and the lamina propria comprises loose connective tissue, blood vessels, and nerves. Sparse smooth muscle fibers, often discontinuous, represent the muscularis mucosa. Distinctly, the muscularis propria (detrusor muscle) is formed by smooth muscle bundles running irregularly in all directions. Thus, these layers are expected to present very different stiffness and therefore very different microenvironments to the cells within [13]. However, mechanical measurements distinguishing bladder layers have not yet been performed to our knowledge. In this work, we measured the micromechanical properties of bladder tissues from bladder cancer patients using AFM. Specifically, we obtained the Young’s modulus of both the mucosal (urothelium, subjacent lamina propria, and sparse muscular fibers of the muscularis mucosa) and muscularis propria layers of the bladder wall.

## 2. Materials and Methods

### 2.1. Patients and Tissue Samples

Fresh BlCa tissues (*n* = 5, newly diagnosed) were collected, along with adjacent normal tissue samples, from patients who underwent radical cystectomy in 2021 at the Portuguese Oncology Institute of Porto (IPO-Porto). All tissue samples were fresh-frozen at −80 °C and subsequently cut in a cryostat for AFM assay. The respective twin formalin-fixed paraffin-embedded (FFPE) tissue fragments were processed at the Department of Pathology and later evaluated by a dedicated uropathologist for routine histopathological examination, including grading and pathological staging, according to the most recent 2016 WHO classification. Mucosa and muscularis propria were marked (on hematoxylin and eosin (H&E) sections) by the uropathologist on replicates of each tested specimen. Using ImageJ, we then measured the thickness in five different locations of each layer and measured the area fraction of smooth muscle on each layer by automatic image thresholding. In addition, relevant clinical data were collected from clinical charts (Table 1). All patients were treated by the same multidisciplinary team of professionals. Patients were enrolled after informed consent. This study was approved by the institutional review board (Comissão de Ética para a Saúde) of IPO-Porto (CES. 92/021).

### 2.2. Tissue Samples Preparation for Atomic Force Microscopy

**Figure 1:**
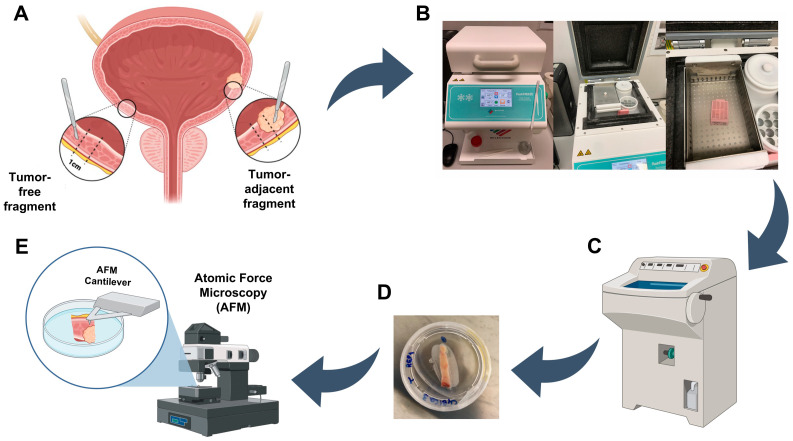
Tissue samples preparation for atomic force microscopy analysis. (**A**) Bladder tissue excision of tumor-adjacent and tumor-free fragments. (**B**) Tissue fragments storage at −80 °C until AFM usage. (**C**) Tissue fragments thinning and preparation for AFM. (**D**) Sample glued in a tissue culture dish. (**E**) Sample immersed in PBS ready for AFM measurements. Created with BioRender.com.

A schematic representation that resumes the optimized protocol for tissue sample preparation for atomic force microscopy (AFM) is represented in Figure 1. Briefly, post-excision, the cystectomy/cystoprostatectomy specimen was maintained in an appropriate container for no more than 2 h at room temperature (RT) until an expert uropathologist performed the first macroscopic evaluation (Figure 1A). Before formalin fixation, a transverse complete section of the bladder wall (~1 cm width) was performed representing all layers (mucosa, muscularis, and perivesical adipose tissue), both through the tumor area (representing the transition to normal mucosa) and through macroscopically normal urothelial mucosa away from the tumor. The fragment was placed in a tissue cassette, and immediately snap-frozen (−80 °C) in 100% ethanol in a FlashFREEZE apparatus (Milestone Medical) (Figure 1B). After this, frozen tissues were immediately transported to the Tumor Biobank and stored at −80 °C until further use.

On the day of the AFM experiment, tissue samples were removed from −80 °C and dissected into ~5 mm thick through-thickness slices using a scalpel blade. Then, tissue slices were included in a cryomold with optimal cutting temperature compound (OCT) and thinned out in a cryostat until the measuring surface was entirely smooth and tissue slices were 1–3 mm thick (Figure 1C). To attach the tissue slices to the 40 × 11 mm tissue culture dishes (TPP Techno Plastic Products, Cat. 93040), cyanoacrylate adhesive glue was used, and tissue slices were placed on top of the glue layer in the dish using tweezers (Figure 1D). After allowing the glue to dry sufficiently for securing the tissue slice, phosphate-buffered saline (PBS) buffer was added to cover the sample completely until AFM analysis (Figure 1E).

### 2.3. Atomic Force Microscopy

Atomic force microscopy (AFM) was applied to determine the mechanical properties of the mucosa and muscularis propria layers for both normal and cancerous tissue samples. Corning dishes containing through-thickness bladder samples were put in a HEPES-Ringer buffer at 37 °C temperature. We then measured the mechanical properties with a NanoWizard III (JPK, Berlin, Germany), using a 5 µm colloidal probe (CP-qp-CONT-BSG-A, NanoAndMore GmbH, Wetzlar, Germany). The spring constant of each probe was measured before the experiments using Bruker’s calibration procedure (Bruker, Santa Barbara, CA, USA). The force constant values ranged from 0.11 to 0.15 N/m. Force curves were recorded with a scan rate of 1 Hz, travel range of 15 µm, tip velocity of 5 µm/s, and a force trigger setpoint of 0.8 nN (indentation depths < 2 µm). Sixty-four curves were acquired over an area of 40 × 40 µm^2^ in the form of force maps. For each layer, three force maps were recorded at different locations, for a total of 192 curves per layer, hence ~384 per tissue. Data were analyzed using JPK Data Processing Software (JPK, Berlin, Germany) to assess the Young’s moduli of each indented layer. We have used the Hertz’s contact model for a spherical indenter to fit the extended curves [14,15]:

$$F^{2/3}={\left(\frac{4}{3}\frac{E}{{\left(1-v\right)}^{2}}R^{1/2}\right)}^{3/2}i$$

where *F* represents the indentation force, *i* is the indentation, *R* is the sphere radius, *υ* is the Poisson’s ratio (assumed as 0.5), and *E* is the Young’s modulus. The samples were sufficiently thick (∼500 μm) compared to the indentation depths (∼1 μm). All curves not in conform with typical indentation–force curves were manually removed.

### 2.4. Statistical Analysis

All statistical analyses and plots generation were performed using Prism 9 (GraphPad Software, San Diego, CA, USA). Mann–Whitney tests were used for testing the equality of means in two groups. A two-way analysis of variance (ANOVA) was used, followed by Tukey’s post hoc test for multiple comparisons of the obtained values. All data are presented as the mean ± std. Data with *p* < 0.05 are considered significant.

## 3. Results

### 3.1. Evaluation of Clinicopathological Parameters & Quantification of Smooth Muscle Fraction

Enrolled patients were all male, with a median age of 73 years old (Table 1). All patients presented invasive high-grade bladder tumors (MIBC) at the time of surgery, and pathological stages ranged from pT3 to pT4. Most patients (*n* = 4/5) also presented concomitant prostate cancer. Only one patient performed neoadjuvant therapy (e.g., gemcitabine + cisplatin) and showed residual tumor in the bladder; although for our analysis, no significant differences were observed for this patient when compared with the others.

**Figure 2:**
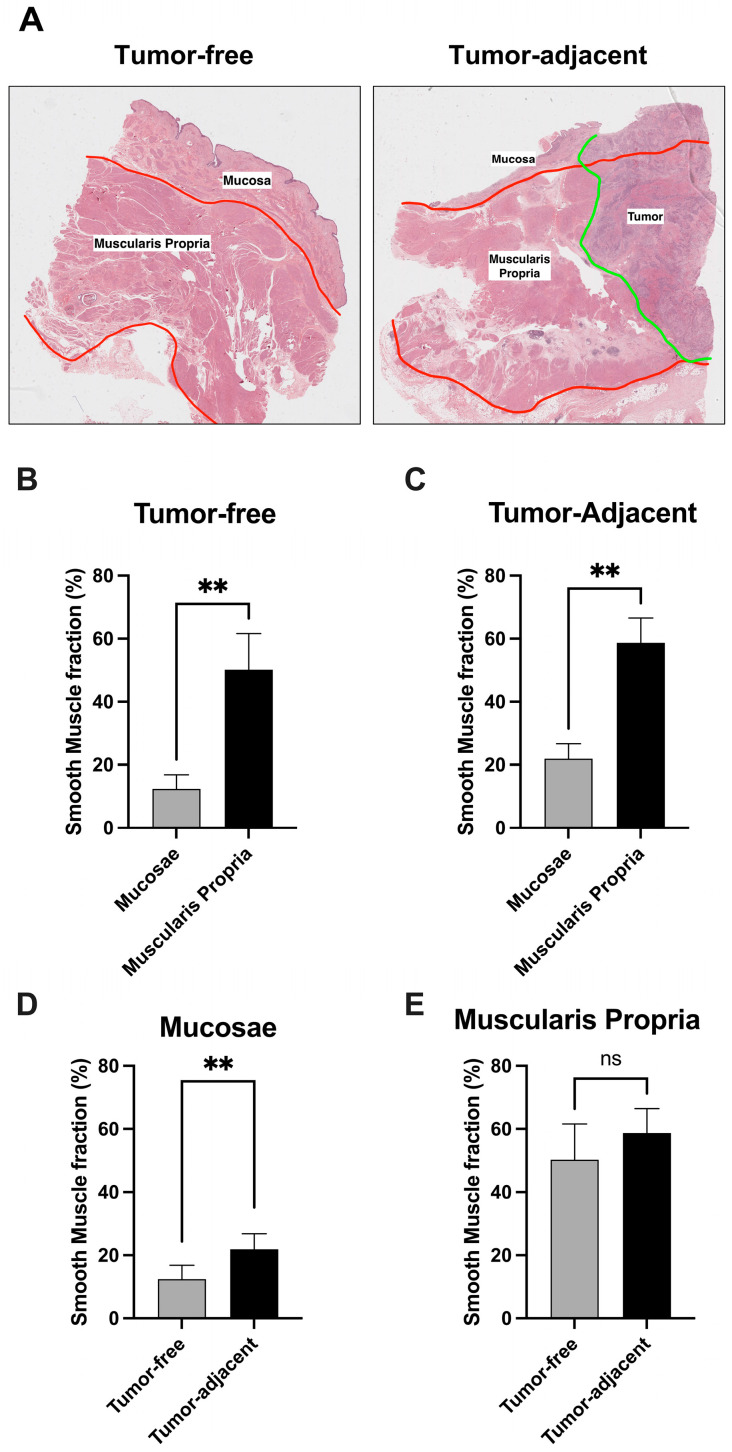
(**A**) Hematoxylin and eosin (H&E) staining of the tumor-free and tumor-adjacent bladder tissue specimens. (**B**,**C**) Comparison of smooth muscle fraction between tumor-free and tumor-adjacent tissue samples. (**D**,**E**) Comparison of smooth muscle fraction between mucosae and muscularis propria bladder layers. ** *p* < 0.001, ns: non-significant.

Using the H&E staining of the tissue specimens we have performed the quantification of smooth muscle fraction in the different bladder layers of the different samples (Figure 2A). Overall, we observed that the smooth muscle fraction was significantly increased in the muscularis propria layer when compared with the mucosae, as expected (*p* ** < 1 × 10^−2^, Figure 2B,C). Interestingly, we have also observed that the smooth muscle fraction is significantly increased in the mucosae of tumor-adjacent samples, when directly compared with tumor-free samples (*p* ** < 1 × 10^−2^, Figure 2D,E).

### 3.2. Increased Stiffness in Tumoral Tissue in Both Mucosa and Muscularis Propria

**Figure 3:**
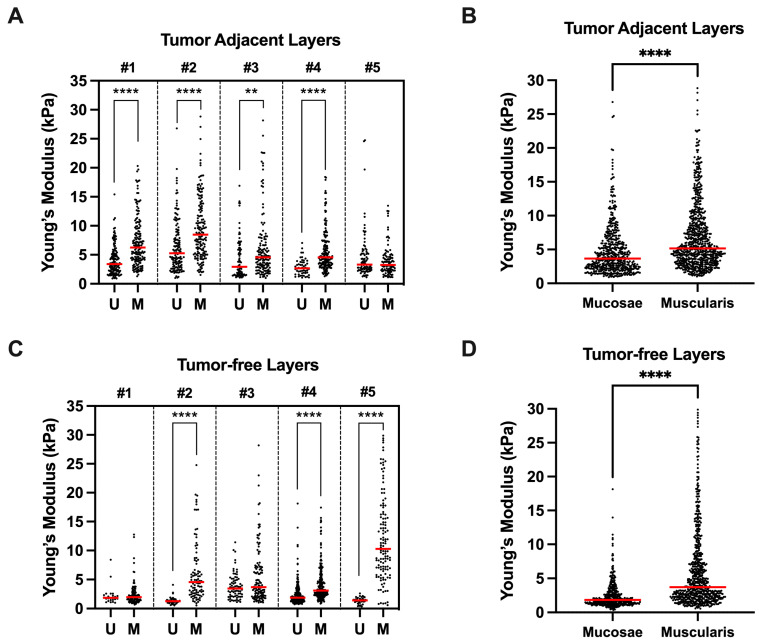
(**A**,**C**) Comparison between bladder wall layers (mucosae (U) vs. muscularis propria (M)) Young’s moduli for each patient according to differently located tissue samples (tumor-adjacent or tumor-free). (**B**,**D**) Mean Young’s moduli of all mucosae vs. muscularis propria measurements according to differently located tissue samples. ** *p* < 0.001, **** *p* < 0.0001.

All patients presented tumor-adjacent tissues with higher Young’s moduli in the muscularis propria region compared with the mucosa region values (Figure 3A). The average Young’s modulus in tumor-adjacent specimens was significantly higher in the muscularis propria than in the mucosa (*p* **** < 1 × 10^−4^, Figure 3B). Patient #5 did not recapitulate this difference in the tumor-adjacent specimens. On histological analysis, the tumor was found to be highly necrotic throughout all depths of invasion, explaining the absence of differences in stiffness. Similarly, the tumor-free specimens had significantly higher Young’s moduli in the muscularis propria than in the mucosa (*p* **** < 1 × 10^−4^, Figure 3D). Patients #1 and #3 did not present significant differences in the tumor-free specimens.

**Figure 4:**
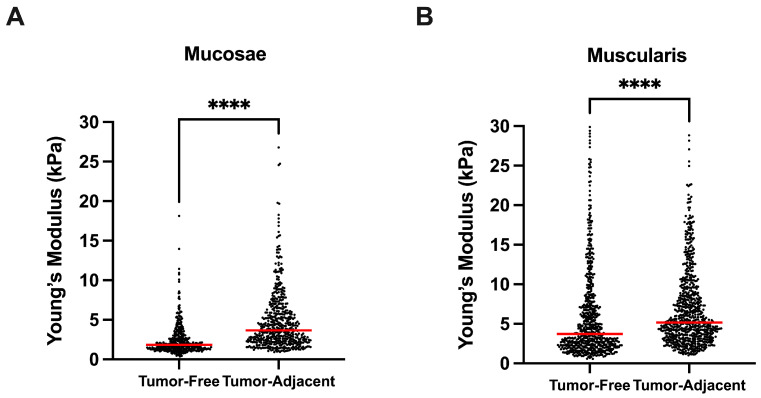
Comparison between bladder tumor-free vs. adjacent tissues, in mucosae (**A**) and muscularis propria (**B**) bladder wall layers. **** *p* < 0.0001.

Both mucosa and muscularis propria stiffness was increased in tumor-adjacent tissues. The mucosa had a significantly higher Young’s modulus in tumor-adjacent tissues than in tumor-free tissues (p **** < 1 × 10^−4^, Figure 4A). Likewise, the muscularis propria had a significantly higher Young’s modulus in tumor-adjacent tissues than in tumor-free tissues (p **** < 1 × 10^−4^, Figure 4B).

## 4. Discussion

Since BlCa therapeutic landscape did not suffer significant improvements in recent years, excluding the approval of immune checkpoint inhibitors for metastatic cases, the scientific community became more interested in exploring other mechanisms that could be crucial for these neoplasms. Indeed, current therapeutic options for BlCa are somehow limited. While the first-line treatment for most BlCa cases is the surgical removal of the tumor or the whole bladder, many patients also fulfill a therapeutic drug-based plan as part of their treatment plan, which depends on tumor stage and grade as other factors. These include BCG or mitomycin C bladder instillations for patients with NMIBC disease or systemic NAC therapies for MIBC cases. However, the high recurrence rate or the inability to fulfill treatment plans due to associated comorbidities is still a problem. Thus, there is an urgent need to find specific mechanisms that could be crucial for the basis of these neoplasms, which might allow for better treating those patients that do not benefit from current therapeutic options. This opens the road to studying different subjects, such as the mechanobiology of bladder cancer cells.

This pilot study tested the hypothesis that the bladder wall presents altered stiffness not only through the bladder wall’s different layers, but also in tumor-adjacent regions. As previously stated, once bladder tumors reach the muscularis propria, the overall prognosis becomes poorer. The molecular, genetic, and epigenetic basis of BlCa carcinogenesis can help explain why some tumors acquire more invasive properties than others, but the mechanical properties of cancer cells and of the surrounding tissues could reveal important mechanisms for tumor invasion to actually occur.

According to the American Joint Committee on Cancer (AJCC) guidelines, we consider the mucosa to be composed of the urothelium and the sub-urothelial connective tissue of the lamina propria [16]. The mucosa layer displays the innermost transitional epithelium with coats of epithelial cells working as a barrier. The lamina propria, which is fundamentally composed of connective tissue, in humans also contains a more ill-defined muscular layer, the muscularis mucosa, with dense irregular connective tissue whose major cell type is fibroblasts; and the tunica submucosa, a relatively thick layer of loose connective tissue rich in collagen fibers, blood, lymphatic vessels and nerve fibers [17,18]. The muscularis constitutes the detrusor muscle and is a smooth muscle layer with coats oriented primarily in the longitudinal and circumferential directions. This layer also contains collagen and other ECM constituents. Indeed, our results correlate well with this description of the bladder wall layers: whereas the muscularis propria presented a considerable % of quantified smooth muscle, the mucosa layer presented a significant decrease in this parameter, although not a complete absence. This may possibly correspond to the presence of the above-mentioned muscularis mucosa in the analyzed cases. Moreover, we observed that tumor-adjacent regions presented a significantly higher % of smooth muscle in mucosae than tumor-free regions among the different conditions. This may suggest that the tumors promote the development of a more “muscle-like” structure in the mucosae region surrounding the neoplastic lesion. Indeed, even though this finding needs to be further explored, some authors suggest that cancer cells infiltrated in the musculature have an enhanced capacity to metastasize or develop therapy resistance, which may indicate that, in order to do that, cancer cells need to stimulate the changing of surrounding tissue towards a “muscle-like” environment [19].

Our findings also confirmed that tissue stiffness depends on both tumor proximity and bladder layer. Consistent with other studies, tumor-adjacent tissues had higher stiffness [20,21]. However, some studies also reported lower stiffness in cancerous tissues [22]. Altered stiffness in tumor-adjacent tissues may be related to the uncharacteristic crosslinking between ECM constituents near the tumor [23]. In pathology, tumor-associated stroma deposition is a well-known phenomenon, histologically recognized by pathologists as desmoplasia. Increased desmoplasia correlates with increased consistency of tumor masses and a whitish coloration. The tumor-associated stroma is part of the tumor microenvironment and is known to interact with tumor progression. There are studies showing that increases in collagen content in cancerous tissues are implicated in the progression from non-muscle-invasive to muscle-invasive bladder cancer [11,24]. Our findings indicate similar smooth muscle content between tumor-adjacent and tumor-free samples. Nonetheless, in this study, we did not measure collagen content since a staining method would be required. Future studies should address the collagen content as well.

Ghasemi et al. showed that ECM stiffness was higher in tumor-adjacent in both newly diagnosed and recurrent BC patients that went transurethral resection [25]. Recurrent patients presented higher stiffness than the newly diagnosed. They reported tissue stiffness between 5 and 15 kPa but not referring which AFM tips were used in their study. In comparison, our average Young’s moduli were lower and below 6 kPa. This may be due to the fact that we have used large 5 μm colloidal probes. Importantly, the use of large colloidal probes (over 1 μm radius) better represents the mechanical stimuli that cells experience in vivo, compared to nanometer size unmodified probe tips [26]. In this study, we also distinguished stiffness between layers mucosa and muscularis propria. Moreover, as seen in the work of Morales-Orcajo et al., our tested muscularis propria layers also presented 50–60% of smooth muscle [27].

One of the major potential applications in the characterization of mechanical properties of human tissues is the unprecedented opportunity to carry out patient-specific studies on the course of disease by using organoids or organ-on-chip systems. By constructing those systems with biological information closest to in vivo conditions and comparing the clinical and in vitro data patterns, we may open the way to finding new early-stage biomarkers, tracking the development of the disease, and developing individualized therapeutic treatment regimens. Moreover, using the mechanical properties of tissues in organoid-like systems would help to reduce the use of in vivo experimentation, surpassing the ethical issues which those tests imply.

One limitation of the present study is that AFM indentations were performed on scan areas with fat, smooth muscle, and other ECM constituents: all presenting distinct stiffness. Our specimens were also relatively thick, which sometimes compromised the AFM data acquisition since the engaging surface was not flat. These limitations led to moderate to high data dispersion in the same scan area. Further, this study used a reduced number of samples. Future studies should distinguish the measured ECM constituents and use larger cohorts. The bladder wall can stretch up to 3–4 times its initial length during filling [28]. The deformations applied to the samples during the AFM experiments were small compared to the physiological levels that the tissues likely experience in vivo. Future studies should test the hypothesis if our results hold in stretched specimens.

## 5. Conclusions

In this study, we provide insights, through AFM measurements, about the stiffness of both mucosa and muscularis propria bladder wall layers, and we show that the presence of tumor in surrounding mucosae may change its smooth muscle content. The quantitative assessment of the bladder wall stiffness range here presented provides essential data for future research on BlCa, to modulate cell culture-based in vitro and in vivo experiments, and for understanding how cancer cells invade through the different bladder layers.

## Figures and Tables

**Table 1:** Clinical and histopathological parameters of bladder cancer patients included in the study.

**Patient** **ID**	**Gender**	**Age**	**Grade**	**Pathological** **Stage**	**Invasion of Organs and** **Adjacent Structures**	**Metastasis**	**Lesion** **Size** (**cm**)	**Bladder Size** (**cm**)	**Concomitant** **Neoplastic Disease**	**Neoadjuvant** **Treatment**
1	M	69	IHG	pT4	No	Yes	7 × 7	7.8 × 8 × 4.9	PCa	No
2	M	79	IHG	pT4	Prostate and seminal vesicle	No	3.4 × 2.1	4.4 × 3.7 × 2.8	PCa	No
3	M	82	IHG	pT3a	No	No	3.3 × 2.3	7 × 5.5	PCa	No
4	M	61	IHG	pT3	No	No	6 × 5.5	6.5 × 5.5	PCa	Gencitabine + cisplatine
5	M	76	IHG	pT4a	Urethra	No	4.7 × 4.2	8 × 7.5	No	No

M—male; IHG—invasive high-grade; PCa—prostate cancer.

## Data Availability

Not applicable.
